# Impact of Continuous Axenic Cultivation in *Leishmania infantum* Virulence

**DOI:** 10.1371/journal.pntd.0001469

**Published:** 2012-01-24

**Authors:** Diana Moreira, Nuno Santarém, Inês Loureiro, Joana Tavares, Ana Marta Silva, Ana Marina Amorim, Ali Ouaissi, Anabela Cordeiro-da-Silva, Ricardo Silvestre

**Affiliations:** 1 Parasite Disease Group, IBMC - Instituto de Biologia Molecular e Celular, Universidade do Porto, Porto, Portugal; 2 Departamento de Ciências Biológicas, Faculdade de Farmácia, Universidade do Porto, Porto, Portugal; 3 INSERM, UMR, CNRS 5235, Université Montpellier II, Montpellier, France; National Institutes of Health, United States of America

## Abstract

Experimental infections with visceral *Leishmania* spp. are frequently performed referring to stationary parasite cultures that are comprised of a mixture of metacyclic and non-metacyclic parasites often with little regard to time of culture and metacyclic purification. This may lead to misleading or irreproducible experimental data. It is known that the maintenance of *Leishmania* spp. *in vitro* results in a progressive loss of virulence that can be reverted by passage in a mammalian host. In the present study, we aimed to characterize the loss of virulence in culture comparing the *in vitro* and *in vivo* infection and immunological profile of *L. infantum* stationary promastigotes submitted to successive periods of *in vitro* cultivation. To evaluate the effect of axenic *in vitro* culture in parasite virulence, we submitted *L. infantum* promastigotes to 4, 21 or 31 successive *in vitro* passages. Our results demonstrated a rapid and significant loss of parasite virulence when parasites are sustained in axenic culture. Strikingly, the parasite capacity to modulate macrophage activation decreased significantly with the augmentation of the number of *in vitro* passages. We validated these *in vitro* observations using an experimental murine model of infection. A significant correlation was found between higher parasite burdens and lower number of *in vitro* passages in infected Balb/c mice. Furthermore, we have demonstrated that the virulence deficit caused by successive *in vitro* passages results from an inadequate capacity to differentiate into amastigote forms. In conclusion, our data demonstrated that the use of parasites with distinct periods of axenic *in vitro* culture induce distinct infection rates and immunological responses and correlated this phenotype with a rapid loss of promastigote differentiation capacity. These results highlight the need for a standard operating protocol (SOP) when studying *Leishmania* species.

## Author Summary

Protozoan of the genus *Leishmania* undergo several developmental transitions during its life cycle. *Leishmania* alternates between two morphologically distinct forms, promastigotes (insect stage) and amastigotes (vertebrate stage). Most of the available information about *Leishmania* spp. has been obtained from studying *in vitro* cultured promastigotes, an excellent experimental model for the different developmental stages present in the insect vector. Although promastigotes are grown in a controlled environment, the maintenance of long term culture results in loss of virulence, which can lead to a misinterpretation and often contradictory experimental results. It is then of great interest to unravel the defects arising from sustained axenic parasite culture in laboratory settings. The authors demonstrate a correlation between the maintenance of parasite culture with a growing defect of the promastigote form to differentiate in the mammalian amastigote form. This research provides a biological explanation for the loss of virulence due to sustained parasite culture and discusses the impact for all experimental work done with visceral *Leishmania* species.

## Introduction

Protozoan parasites of the genus *Leishmania* undergo several developmental transitions during their life cycle. Ingestion of infected macrophages during a blood meal by the sandfly vector leads to the release of intracellular amastigotes into the vector's midgut. This abrupt change in environment induces the transformation into extracellular procyclic promastigotes. The procyclic form within the vector midgut replicates and ultimately transforms into virulent metacyclic promastigotes, in a complex process that encompass parasite migration towards the upper gut of sandfly vector [1]. In laboratory conditions, it is possible to achieve indefinite promastigote growth outside the sandfly using several established media; the procyclic forms correspond to promastigotes in exponential phase of growth that will eventually pass into a stationary phase, a fraction of these stationary parasites differentiates into the metacyclic form, with properties resembling those of sand fly promastigotes [2], [3]. Although stationary-phase promastigotes with undefined *in vitro* passages are commonly used without limitations, it has been demonstrated that continuous culture over time induces loss of virulence. In fact, long-term *in vitro* culture of promastigotes was one of the first empirical approaches to efficiently identify parasite virulence genes leading to the experimental development of attenuated strains [4]. Similarly, long-term *in vitro* growth of drug-resistant parasites was suggested to mediate a loss of the resistance phenotype [5]. This can be due to either loss of virulence factors induced by the lack of a survival pressure or due to disadvantageous adaptations to the media resulting in phenomena similar to clonal selection [6]. Either way, alterations in the physiology of the parasite that are induced by long-term growth in these media may lead to misinterpretation and contradictory results. Thus, one must carefully consider the influence of the different laboratorial factors in order to minimize these variables.

The current study is based upon the hypothesis that maintenance of *Leishmania* spp. in axenic *in vitro* culture results in a progressive loss of virulence quickly generating a significant bias towards the experimental data. We have compared the *in vitro* and *in vivo* infections and focused on the influence that axenic parasite growth and long-term maintenance can have on *in vitro* infections outcome. Our results demonstrate, for the first time, that the loss of virulence caused by the maintenance of axenic promastigotes in culture can be the result of a growing inability to differentiate into amastigote forms. Moreover, the induction of differentiation from promastigote to amastigote and then back to promastigote forms both *in vitro* and *in vivo* was capable to restore parasite virulence. Overall, our study demonstrated the need of a standard operating protocol (SOP) to study visceral *Leishmania* spp. highlighting the crucial importance for proper control of parasite cultures in studies focusing on the mammalian stage, such as drug development or vaccine trials.

## Materials and Methods

### Animals and parasites

Ten to twelve-week-old female Balb/c mice were obtained from Instituto de Biologia Molecular e Celular (IBMC; Porto, Portugal) animal facilities. Under laboratory conditions, the animals were maintained in sterile cabinets and allowed food and water *ad libitum*. Animal care and procedures were in accordance with institutional guidelines. All conducted experiments were done in accordance with the IBMC.INEB Animal Ethics Committee and the Portuguese Veterinary Director General guidelines. RS has an accreditation for animal research given from Portuguese Veterinary Direction (Ministerial Directive 1005/92). A cloned line of virulent *L. infantum* (MHOM/MA/67/ITMAP-263) was grown at 26°C in RPMI 1640 medium (Lonza, Swtzerland) supplemented with 10% heat-inactivated Fetal Bovine Serum - FBS (Lonza, Switzerland), 2 mM L-glutamine, 100 U/ml penicillin, 100 mg/ml streptomycin and 20 mM HEPES buffer. The MHOM/MA/67/ITMAP-263 clone (zymodeme MON-1) was originally isolated from the bone marrow of a human patient in Morocco and cloned by micromanipulation. In some experiments, a previously uncharacterized field attenuated *L. infantum* strain was used (species confirmed by pteridine reductase 1 sequencing and currently under ongoing characterization in our laboratory). To minimize the possibility of clonal bias, we have performed three independent recoveries of parasite from Balb/c mice for these experiments. All cultures were initiated at 10^6^ parasites/ml and passed each 5 days. Promastigote to amastigote differentiation was achieved by culturing 10^7^ stationary phase promastigotes/ml at 37°C in a cell free culture medium (MAA20) [7]. Amastigote to promastigote differentiation was performed by culturing 10^7^ axenic amastigotes/ml in complete RPMI medium for 4 days at 27°C. In alternative, spleens of infected Balb/c mice were placed in similar culture conditions for 7 days.

### Ficoll density purification assay

Metacyclic promastigotes were purified from cultures with 3, 5 or 9 days or from 5-day cultures with 4, 21 and 31 (P4, P21 and P31) *in vitro* passages by Ficoll density gradient, as previously described [8]. Briefly, 6 ml of 40% Ficoll was overlaid by 6 ml of 10% Ficoll in RPMI base. Then, 6 ml of PBS containing 1.2×10^9^ parasites was placed at the top of the Ficoll gradient. The step gradient was centrifuged for 10 minutes at 370 g at room temperature without brake. The metacyclics promastigotes were recovered from the layer between 0% and 10% Ficoll solution. Metacyclic promastigotes, identified by morphological criteria, *i.e.*, short and slender with a long flagellum twice the body length using phase contrast on a Nikon Eclipse 80i.

### qPCR analysis

Total RNA was isolated from cells with the Trizol® reagent (Invitrogen, Barcelona, Spain), according to the manufacturer's instructions. Briefly, parasites were washed with ice-cold phosphate-buffered saline (PBS), harvested and homogenized in 800 µl of Trizol by pipetting vigorously. After addition of 160 µl of chloroform, the samples were vortexed, incubated for 2 min at room temperature and centrifuged at 12.000 g, for 15 min, at 4°C. The aqueous phase containing RNA was transferred to a new tube and RNA precipitated with 400 µl of isopropanol for at least 10 min at room temperature. Following a 10 min centrifugation at 12.000 g, the pellet was washed with 1 ml of 75% ethanol and resuspended in 10 µl of 60°C heated RNase free water. The RNA concentration was determined by using a Nanodrop spectrophotometer (Wilmington, DE, USA) and quality was inspected for absence of RNA degradation or genomic DNA contamination, using the Experion RNA StdSens Chips in the ExperionTM automated microfluidic electrophoresis system (BioRad Hercules, CA, USA). RNA was stored at −80°C until use. RT was performed with equal amounts of total extracted RNA (1 µg) obtained from parasites recovered from different experimental conditions by using Superscript II RT (Gibco BRL) and random primers (Stratagene). Real-Time quantitative PCR (qPCR) reactions were run in duplicate for each sample on a Bio-Rad My Cycler iQ5 (BioRad, Hercules, CA, USA). Primers sequences were obtained from Stabvida (Portugal) and thoroughly tested. qPCR was performed in a 20 µl volume containing 5 µl of complementary cDNA (50 ng), 10 µl of 2× Syber Green Supermix (BioRad, Hercules, CA, USA), 2 µl of each primer (250 nM) and 1 µl H_2_O PCR grade. Specific primers for histone H4 (forward: 5′ ACACCGAGTATGCG -3′; reverse: 5′- TAGCCGTAGAGGATG-3′; LinJ35.1400 histone H4: Gene ID 5073031), Small Hydrophilic Endoplasmic Reticulum-associated Protein (SHERP) (forward: 5′ CAATGCGCACAACAAGAT -3′; reverse: 5′- TACGAGCCGCCGCTTA-3′; LinJ23.1190 SHERP: Gene ID 5069222) and rRNA45 (forward: 5′CCTACCATGCCGTGTCCTTCTA -3′; reverse: 5′- AACGACCCCTGCAGCAATAC -3′) [9] were used for amplification. After amplification, a threshold was set for each gene and cycle threshold-values (Ct-values) were calculated for all samples. Gene expression changes were analyzed using the built-in iQ5 Optical system software v2.1 (Bio-Rad laboratories, Inc). The results were normalized using as reference gene the rRNA45 rRNA sequence [9].

### Viability analysis

Purified and non-purified promastigotes at a density of 10^5^/ml were washed and suspended in Annexin V binding buffer. Parasites were incubated at room temperature for 15 minutes with AnnexinV-Cy5 (BD Pharmingen, San Diego, CA) and 7-AAD (Sigma). Parasites subjected to Ultra Violet light during 30 minutes and kept in culture for 4 hours were used as positive control. In amastigote differentiation, 2×10^6^ cells with 1 µM of propidium iodide (PI) were used. Data were collected in a BD FACScalibur cytometer (20.000 gated events) and analyzed by FlowJo software (Ashland, OR).

### Promastigote CFSE-labeling

Purified and non-purified promastigotes (6×10^7^/ml) were washed two times, suspended in PBS containing 5 µM of carboxyfluorescein succinimidyl ester (CFSE) (Invitrogen Molecular probes, Eugene, Oregon) and incubated at 37°C for 10 minutes. Labeled parasites were washed, incubated at 4°C for 5 minutes. Parasites were washed again to remove the exceed CFSE dye and suspended in culture medium before proceeding to macrophage infections. For promastigote to amastigote differentiation and proliferation analysis, 10^7^ CFSE-labeled promastigotes were placed on 1 ml of MAA20. Each day, 100 µl of culture added with 1 µM of PI was analyzed by flow cytometry. Axenic amastigotes, identified by the absence of visible flagella and oval shape body, were observed in phase contrast on a Nikon Eclipse 80i.

### *In vitro* macrophage infection

Cell suspension of bone marrow was obtained by flushing the femurs of susceptible Balb/c mice. The cell suspension was cultured in Dulbecco's modified Eagle's medium (DMEM) (Lonza, Switzerland), supplemented with 10% heat-inactivated FBS (Lonza, Switzerland), 2 mM L-glutamine, 100 U/ml penistreptomycin and 1 mM sodium pyruvate. After overnight incubation at 37°C, non-adherent cells were recovered (300 g for 10 min, at room temperature) and cultured in 24-well culture dishes at 2×10^5^cells/ml in supplemented DMEM. For bone-marrow derived macrophages (BMMø) differentiation 10% L-929 cell conditioned medium (LCCM) was added at days 0 and 4. At day 7 of culture, CFSE labeled promastigotes were incubated with the BMMø at a 10∶1 ratio. After 4 hours, infection was stopped the infection rates were determined at 4, 24 and 48 hours post-infection by a BD FACScalibur cytometer and analyzed by FlowJo software. In some experiments, BMMø were infected and submitted to lipopolysaccharide (LPS) stimulus. Briefly, four hours after infection, infection was stopped and 1 µg/ml of LPS (Sigma) added. Twenty-four hours post-infection, BMMø culture supernatants were collected for cytokine quantification by Enzyme-Linked Immunosorbent Assay - ELISA (TNF-α, IL-12p40, IL-6 and IL-10), using commercial sandwich immunoassay kits (Biolegend and BD, San Diego, CA). Also, BMMø were recovered at 24 hours post-infection for surface co-stimulatory markers analysis. Thus, BMMø were stained with CD40-PE and MHCII-APC at 4°C, during 30 minutes in the dark. The cells were then washed in PBS and suspended in 200 µl of PBS-2% FBS. Data were collected by a BD FACScalibur cytometer and analyzed by FlowJo software.

### Animal experiments and parasite quantification

Promastigotes recovered from stationary culture with 4, 21 and 31 *in vitro* passages stationary-culture were collected, washed and suspended in sterile PBS. A volume of 200 µl of PBS containing 10^8^ parasites was injected intraperitoneally. Mice of each group were sacrificed at 56 days post-infection. The parasite burden in the spleen and liver was determined by limiting dilution as previously described [10].

### Statistical analysis

The data was analyzed using the non-parametric Kruskal-Wallis test followed by Dunn posttest for multiple comparisons when necessary.

## Results

### The maintenance of *L. infantum* promastigotes in axenic cultures results in diminished virulence

We started by clarifying our *in vitro* model of *L. infantum* infection in relation to the parasite development stage. *L. infantum* parasites recovered from the spleen of infected Balb/c mice were used to start axenic cultures at a 10^6^ parasites/ml. The first task was to clearly define the culture time frame in which we can recover stationary parasites. Performing basic cell cycle analysis we excluded the use of the parasites until 2 days of culture because there was still significant active division (Fig. S1A). In order to evaluate the infectivity of stationary *L. infantum*, we used CFSE-labeled stationary promastigotes recovered at 3, 5 and 9 days of *in vitro* growth and BMMø as infection cellular target. Our data demonstrate that 3^rd^ culture-day *L. infantum* promastigotes were significantly less infectious when compared with the 5^th^ and 9^th^ days of culture (Fig. S1B). These differences were already observed at 4 hours post-infection indicating a deficient parasite uptake with 3^rd^ culture-day *L. infantum* promastigotes. Intraphagolysosomal adaptation mechanisms do not appear to be involved in the infection differences since similar infection percentages reductions were found between 4 and 24 hours (15.6±0.4 for 3^rd^ culture-day; 15.0±1.2 for 5^th^ culture-day; 18.1±1.1 for 9^th^ culture-day, when comparing 4 with 24 hours post-infection). Many reports now relate virulence with parasites culture viability [11], [12]. In order to lay down the hypothesis that differences in infectivity could be attributed to non-viable parasites, we evaluated the percentages of apoptotic or necrotic parasites by AnnV/7AAD labeling [13]. Nevertheless, no significant differences were found between all culture days (data not shown).

**Figure 1:**
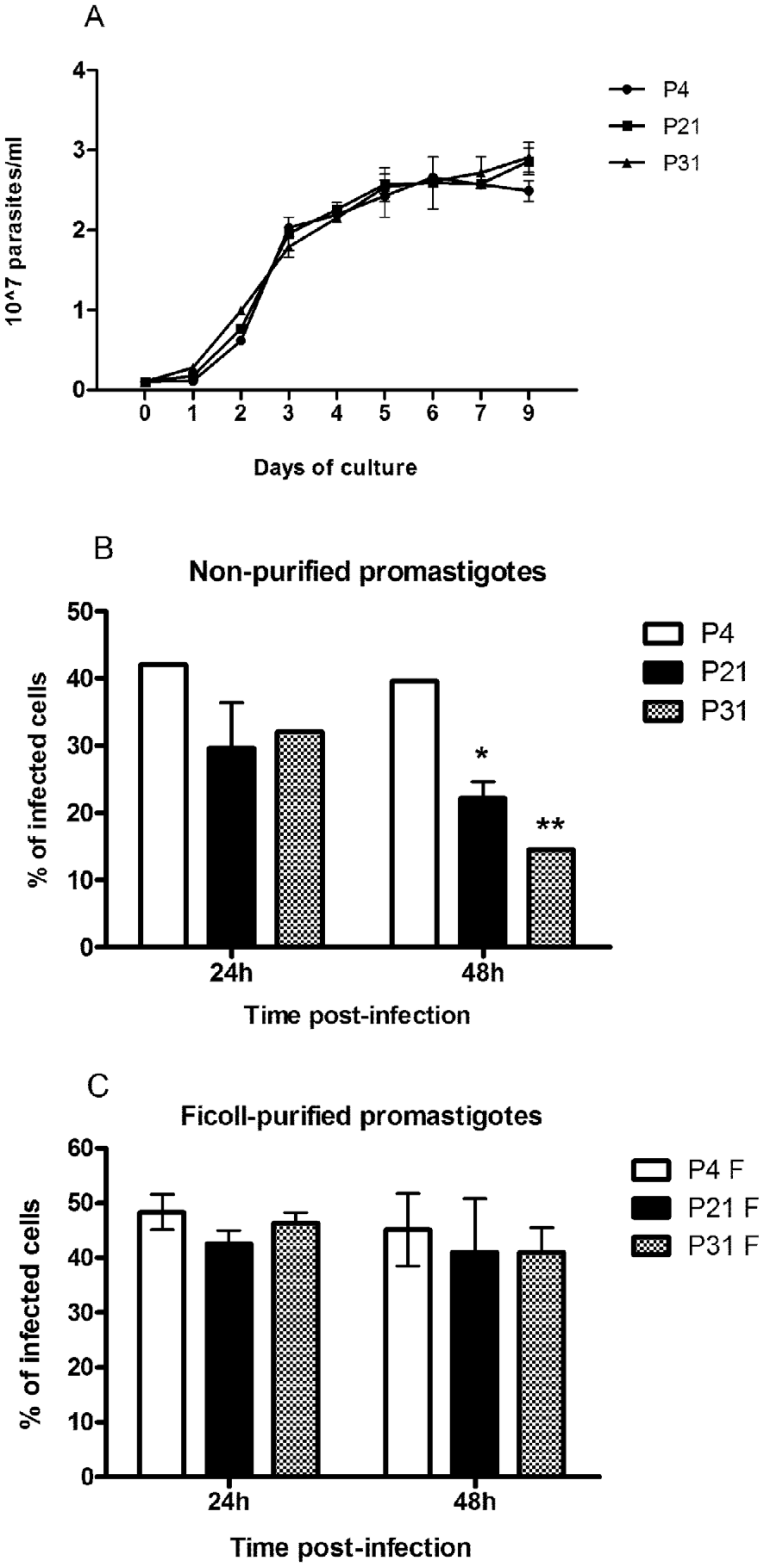
Long-term *in vitro* maintenance of *L. infantum* promastigotes does not alter parasite growth. *L. infantum* growth curves were performed by neubauer chamber counting (**A**). BMMø were infected at a 1∶10 (cell/parasite) ratio with non-purified (**B**) and Ficoll-purified (**C**) promastigotes labeled with CFSE. Data were acquired by FACScalibur cytometer and analyzed by FlowJo software. Three independent experiments were performed; one representative experiment is shown. The mean and standard deviation are shown. *P<0,05, **P<0,01, ***P<0,001 statistical significance relatively to P4.

Several groups have already reported that long-term *in vitro* cultivation (more than 12 months) of *Leishmania* spp. leads to a totally avirulent promastigote population [6], [14]. According to these findings, we decided to evaluate if the sustained maintenance of *L. infantum* promastigotes in axenic culture at shorter time periods lead to distinct BMMø *in vitro* infection rates with distinctive immunologic phenotypes. In order to accomplish this, we maintained *L. infantum* promastigotes recovered from the spleen of infected mice for 4, 21 and 31 passages, which are equivalent to 20, 105 and 155 division events, considering simple exponential growth. The long-term maintenance of *L. infantum* in culture did not modify the promastigote growth behavior (Fig. 1A) neither their viability that was always superior to 90% (data not shown). Taking into account the distinct infection profiles depicted in Fig. S1B, we chose 5-day culture promastigotes to compare infectivity. When non-purified parasite cultures were used to *in vitro* infect BMMø, a marginal but significant loss of infectivity at 48 hours for P21 and P31 when compared to P4 was observed (Fig. 1B). Metacyclic forms have been understood to be the most infective parasite form [8], [15]. Therefore, we enriched the promastigote culture recovered from P4, P21 and P31 in metacyclics recovered by Ficoll density gradient, herein referred as Ficoll-purified promastigotes [16], and analyzed their infectivity in primary BMMø cells (Fig. 1C). When Ficoll-purified metacyclic promastigotes from these cultures were used, differences were abrogated irrespective of the passage used (Fig. 1C). To confirm the enrichment of metacyclic promastigotes in this fraction and as an internal control of our experimental conditions, we analyzed the expression of two genes, SHERP and histone H4 that can be used to evaluate metacyclogenesis. SHERP gene is found to be up-regulated in infective metacyclic promastigotes [17]. On the other hand, higher expression of histone H4 is associated with exponential phase promastigotes [18]. Indeed, the qPCR analysis demonstrated a significant increase in the SHERP mRNA transcripts in Ficoll-purified promastigotes when compared with non-purified parasite cultures (Fig. S2). These results suggested that the maintenance of *L. infantum* in axenic cultures resulted in a virulence leakage affecting their infectivity probably by the loss of metacyclic parasites. Nevertheless, no significant differences were found between the percentage of Ficoll-purified promastigote recovery from each culture (P4: 6.6±0.1%; P21: 3.4±1.6%; P31: 4.0±0.9%).

### Maintenance of long-term axenic *L. infantum* cultures decrease parasite capacity to modulate host cell functions in an inflammatory context

**Figure 2:**
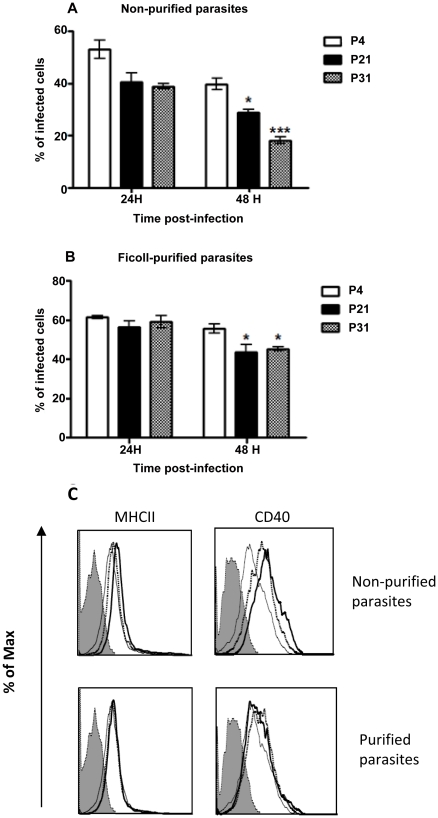
Long-term *in vitro* maintenance of *L. infantum* promastigotes results in loss of virulence *in vitro*. BMMø were submitted to LPS stimulation 4 hours after infection with non-purified (**A**) or Ficoll-purified (**B**) CFSE-labeled promastigotes at a 1∶10 (cell/parasite) ratio. The percentage of infected cells was determined by the number of CFSE-positive cells in a FACScalibur cytometer. Expression of MHCII and co-stimulatory molecule CD40 at 24 hours post-infection. Thick line – P31, dotted line - P4, thin line - LPS and shadded hystogram - isotype control (**C**). Two independent experiments were performed; one representative experiment is shown. The mean and standard deviation are shown. *P<0,05; **P<0,01 statistical significance relatively to P4.

We have above demonstrated that the BMMø infection by *L. infantum* promastigotes depends not only upon the days of culture but is significantly modulated by their axenic culture period. However, we were unable to detect any major changes on macrophage activation status when submitted to *L. infantum* infection (data not shown), which can be explained by the *Leishmania* silent entry mechanism [19]. Therefore, we hypothesized that, when facing an inflammatory stimulus, axenic cultures with high passage number should be less successful in subverting macrophage effector functions being less capable of promoting infection. In order to investigate this hypothesis, we incubated BMMø cells with Ficoll-purified or non-purified parasites from distinct culture periods, which were 4 hours later submitted to LPS stimulation. As before, we observed a decrease of the infection rate with the augmentation of parasite *in vitro* passages (Fig. 2A). This difference was minimized if Ficoll-purified promastigotes were used instead, although it was still statistically significant at 48 hours post-infection (Fig. 2B). LPS stimulation rapidly induces a surface up-regulation of MHCII molecules and co-stimulatory marker CD40. The analysis of these markers demonstrated that high passage number parasites had lower capacities to counteract the LPS activation stimulus (Fig. 2C). Once again, these differences were abrogated when metacyclic-enriched populations were used (Fig. 2C). We have also evaluated the levels of secreted IL-6, IL-12p40 and TNF-α and of the anti-inflammatory IL-10 cytokine. We found that the capacity to control LPS-induced cytokines was variable depending on the number of parasite passages, likely reflecting its distinct virulence. While significant differences were found with P31 parasites in the BMMø secretion levels of IL-6 and TNF-α (Fig. S3A and S3B, respectively), the major modifications were observed at the IL-12p40 and IL-10 levels (Fig. S3C and S3D, respectively). Indeed, P4 parasites were more capable to down-regulate IL-12p40 secretion induced by LPS stimulus, while increasing IL-10 cytokine secretion. This demonstrates that high passage parasites failed to counteract the secretion of pro-inflammatory cytokines induced by LPS in a similar manner. Moreover, if a pro-inflammatory/IL-10 ratio is constructed, a strong correlation was observed between shorter axenic culture maintenance periods and lower pro-inflammatory/IL-10 ratios (Table 1). Since the metacyclic enrichment diminished the differences observed in parasite infection rate and co-stimulatory markers found with stationary-phase promastigotes in different passages, we further investigated whether the cytokine bias was similarly altered. Indeed, the use of Ficoll-purified metacyclic enriched promastigotes, whatever their source, shifted the cytokine environment towards an anti-inflammatory ratio. Although some statistical differences were found between Ficoll-purified promastigotes from different passages, all displayed lower pro-inflammatory/IL-10 ratios when compared with LPS stimulation (Table 1). Overall, these data suggests that when *Leishmania*-infected BMMø are faced with an inflammatory stimulus, there is a specific overall loss of modulatory capacity that seems to be related to the highly immunoregulatory population of metacyclic parasites.

**Table 1:** Ratio of several pro-inflammatory cytokines/IL-10 quantified by ELISA on cell supernatants of 24 hours infected BMMø.

		IL-12p40/IL-10	IL-6/IL-10	TNF-α/IL-10
**Non - purified**	**LPS**	1,15 ± 0,13	3,46 ± 0,14	6,74 ± 0,40
	**LPS P4**	0,80± 0,01^a^^,^ b	0,40 ± 0,003*** a ^,^ b	0,30 ± 0,016*** a ^,^ b
	**LPS P21**	2,41 ± 0,04*	1,39 ± 0,10*	1,09 ± 0,06*
	**LPS P31**	1,95 ± 0,72	1,15 ± 0,13*	0,84 ±0,07*
**Ficoll - purified**	**LPS P4**	0,18 ± 0,00*** c ^,^ d	0,15 ± 0,01*** c ^,^ d	0,09 ± 0,01*** c ^,^ d
	**LPS P21**	0,45 ± 0,02***	0,39 ± 0,01**	0,19 ± 0,03**
	**LPS P31**	0,40 ± 0,01***	0,40 ± 0,01**	0,18 ± 0,01**

The values are expressed in arbitrary units. One representative experiment out of two is shown. The mean and standard deviation are shown.

*P<0.05,

**P<0.01,

***P<0.001 statistical significant relatively to LPS and between passages of culture presented.

a *P < 0.05 between LPS P4 and LPS P21,

b *P < 0.05 between LPS P4 and LPS P31,

c *P < 0.05 between LPS P4 and LPS P21 and,

d *P < 0.05 between LPS P4 and LPS P31. All Ficoll-purified ratios are significantly different (at least *P < 0.05) from the related non-purified population.

### Sustained culture of *L. infantum* promastigotes results in an *in vivo* loss of virulence

**Figure 3:**
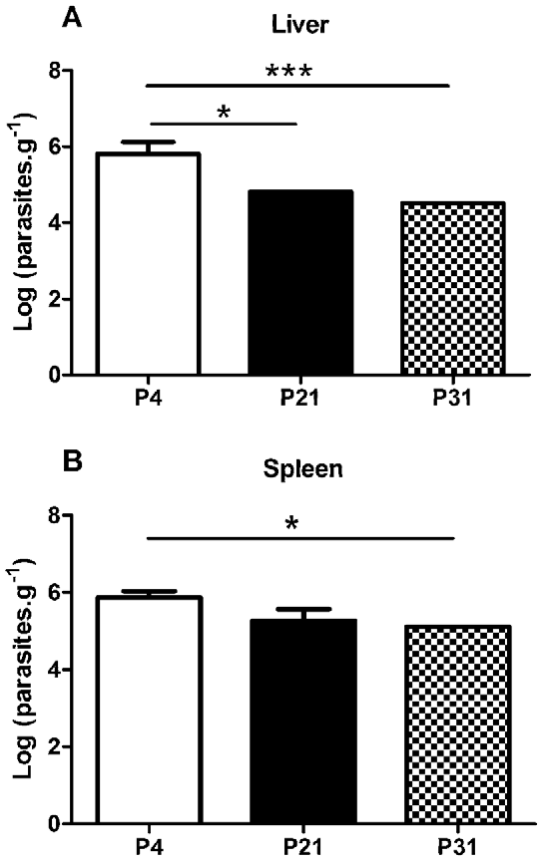
Long-term *in vitro* maintenance of *L. infantum* promastigotes results in loss of virulence *in vivo*. Balb/c mice were infected with stationary phase promastigotes submitted to 4, 21 or 31 successive *in vitro* passages. After 6 weeks post-infection, the parasite load was determined in liver (**A**) and spleen (**B**) by limiting dilution. The mean and standard deviation are shown. *P<0,05; **P<0,01; ***P<0,001.

We have above demonstrated that sustained axenic parasite culture results in a rapid loss of *in vitro* virulence. Previous reports demonstrating an *in vivo* loss of virulence were based on long-term, usually more than 1 year, parasite maintenance [6], [14]. However, we have observed a clear *in vitro* defect after only 21 passages. Therefore, we decided to validate the observed phenotype by performing *in vivo* infections using the susceptible Balb/c mice model. Six weeks after the infectious challenge with non-purified stationary-phase promastigotes recovered from P4, P21 or P31 cultures, a significant difference was found between P4 and high passage number parasite infections in the liver (Fig. 3A). Similarly, we have observed a significant lower parasite burden in the spleen of P31 infected mice that P4 infections (Fig. 3B). These results confirm the observed *in vitro* loss of virulence with parasite culture maintenance.

### Loss of virulence originates from inadequate capacity to differentiate into amastigote forms

**Figure 4:**
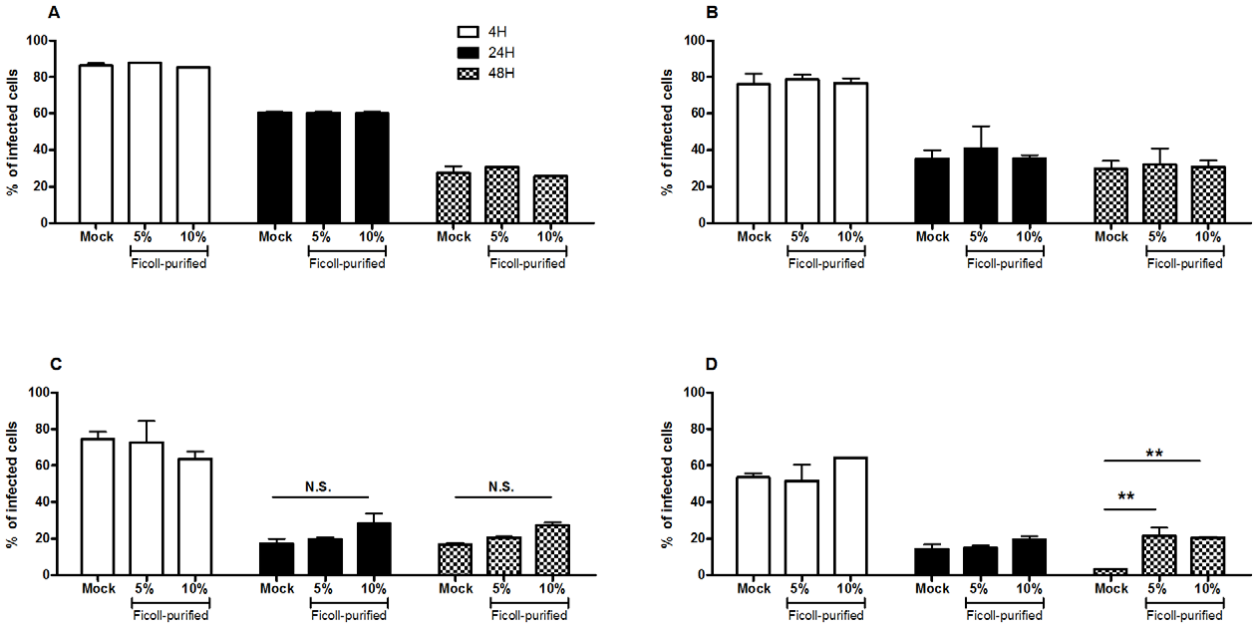
Decreased capacity to infect is not correlated with a loss of metacyclic promastigotes. BMMø were infected at a 1∶10 (cell/parasite) ratio with non-purified promastigotes submitted to 4 (**A**), 21 (**B**) or 31 (**C**) successive *in vitro* passages with 5% or 10% of Ficoll-purified parasites or without (Mock). As a control, BMMø were infected with a naturally attenuated strain in the same conditions (**D**). Data were acquired by FACScalibur cytometer and analyzed by FlowJo software. Two independent experiments were performed; one representative experiment is shown. The mean and standard deviation are shown. **P<0,01.

**Figure 5:**
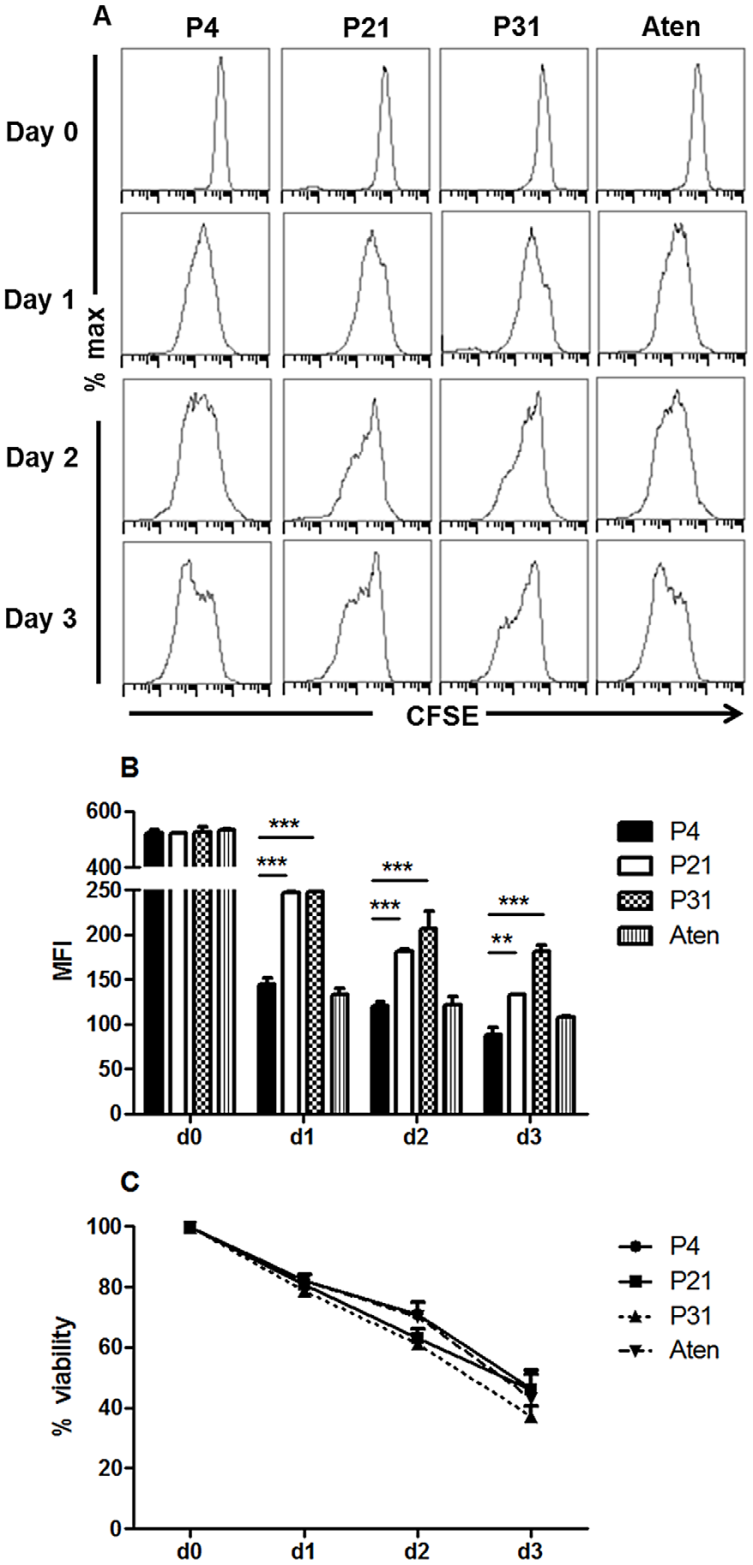
Diminished capacity of long-term cultured *L. infantum* promastigotes to differentiate and proliferate as axenic amastigotes. CFSE-labeled non-purified promastigotes submitted to 4, 21 or 31 successive *in vitro* passages or from a field recovered naturally attenuated strain (Aten) were cultured in MAA20 for 3 days to induce differentiation into the amastigote form. (**A**) Parasite multiplication was followed by FACScalibur quantification of CFSE fluorescence. (**B**) For each time, the mean fluorescence intensity (MFI) was calculated. The mean and standard deviation are shown. **P<0,01; ***P<0,001 (**C**) At the same time, parasite viability was followed by propidium iodide (PI) incorporation.

To elucidate the biological mechanisms that account for the loss of virulence due to long term parasite culture, we started by hypothesizing two major potential reasons: decrease number of metacyclic promastigotes or inadequate differentiation into amastigote forms. The quantification of Ficoll-purified promastigotes described above did not show any significant differences among the passages suggesting similar metacyclic quantities. However, the Ficoll density gradient assay is not a specific and sensible test for the quantification of metacyclic promastigotes in a culture but rather a method for its enrichment. Thus, to evaluate the hypothetical deficit on the generation of metacyclic promastigotes, we have performed *in vitro* infections using BMMø as targets, where we substitute 5% or 10% of non-purified stationary-phase promastigotes from each passage with similar percentages of Ficoll-purified fractions of P4 cultures to increase the total percentage of metacyclic promastigote. As a positive control, we used a naturally attenuated *L. infantum* from which we were unable to recover metacyclic promastigotes. Indeed, although the promastigotes of this *L. infantum* strain presents a similar axenic growth curve (Fig. S4), we were always unable to recover by Ficoll density gradient relevant number of promastigotes (lower that 0.1% of initial culture) from stationary-phase cultures. The quantification of CFSE-positive BMMø demonstrated that increasing the percentage of Ficoll-purified promastigotes did not significantly enhance, at any time point, the percentage of infected BMMø for P4 (Fig. 4A), P21 (Fig. 4B) or P31 (Fig. 4C) promastigotes. However, the opposite was observed with the naturally attenuated strain, where a significant increase of infected macrophages was observed at 48 hours post-infection (Fig. 4D). These results demonstrated that the lack of virulence originated from sustained parasite culture cannot be reverted by the addition of enriched-metacyclic fractions. This excludes a defect in the capacity to generate metacyclic promastigotes as the inherent biological cause for the virulence loss. Therefore, we investigated the potential role of inadequate capacity to differentiate in the amastigote form. CFSE-labeled stationary-phase promastigotes recovered from each passage were placed on MAA20 [7] and followed for three days. We evaluated the promastigote differentiation by light microscopy, axenic amastigotes proliferation by CFSE labeling and overall viability by PI staining. All cultures presented axenic amastigotes-like cells after 3 days of differentiation (data not shown). However, high passage number promastigotes displayed a striking decrease of differentiated cells. To quantify these differences, we have assessed the progressive diminution in the intensity of CFSE staining after the differentiation process. In fact, while P4 promastigotes progressively diminished CFSE fluorescence (Fig. 5A and B), high passage number promastigotes exhibit a severe defect to proliferate as amastigotes forms, as observed in both histogram curves (Fig. 5A) and quantification of mean fluorescence CFSE intensity (Fig. 5B). Moreover, this defect was not correlated with a difference on cell death, since similar percentages of viable parasites were found for all cultures during the differentiation process (Fig. 5C). Interestingly, the naturally attenuated strain did not display any significant change to differentiate when compared with P4 promastigotes, suggesting a distinct mechanism of loss of virulence that is not related with the capacity to generate axenic amastigotes.

### *L. infantum* virulence is restored after full differentiation to amastigote forms

**Figure 6:**
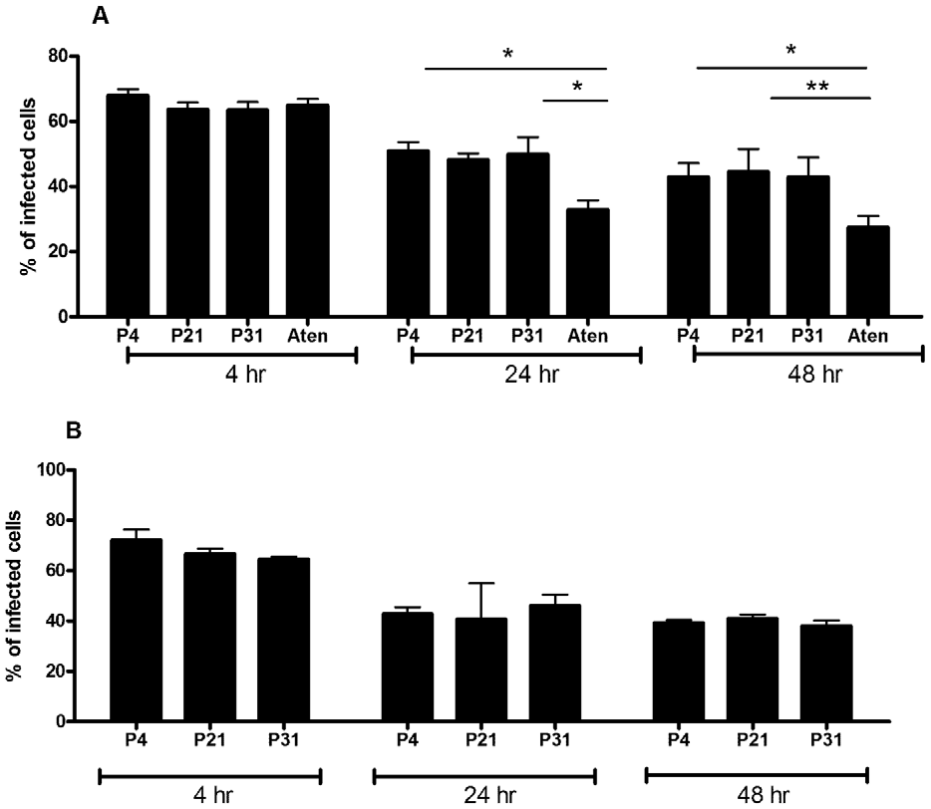
Virulence is recovered after *in vitro* or *in vivo* promastigote-amastigote differentiation process. BMMø were infected at a 1∶10 (cell/parasite) ratio with non-purified promastigotes differentiated from *in vitro* axenic amastigotes (**A**) or *ex-vivo* intracellular amastigotes (**B**). As a control, a naturally attenuated strain (Aten) was submitted to the same experimental conditions. Data were acquired by FACScalibur cytometer and analyzed by FlowJo software. Three independent experiments were performed; one representative experiment is shown. The mean and standard deviation are shown. *P<0,05; **P<0,01.

It is a current empirical methodology to pass *Leishmania* spp. promastigotes in experimental models to maintain virulence. We have hypothesized that the differentiation process from promastigotes to amastigotes forms would select the most virulent parasites in a heterogeneous culture assuring the continuity of competent and adapted parasites. Therefore, to explore this assumption we have differentiated promastigotes from each passage number in amastigotes both in axenic and *in vivo* conditions. Axenic amastigotes were obtained by differentiating promastigotes in MAA20 medium [7] for a period of 3 days. The viable axenic amastigotes were maintained axenically in culture for 10 days, after which were re-differentiated to promastigotes forms. In alternative, we recovered *L. infantum* parasites from the spleen of infected Balb/c mice by allowing amastigote to promastigote differentiation for a period of 7 days. All these promastigotes were sub-cultured for 4 passages and used to infect BMMø. Remarkably, we did not observe any difference between infections whatever the initial parasite source used (Fig. 6A and B). Again, we used as a control the naturally attenuated *L. infantum* strain. Although an increase of virulence was observed after the differentiation protocol (Fig. 6A), when compared to non-differentiated parasites (Fig. 4D), we observed a general lower infection percentage with the exception of 4 hours post-infection. Overall, these results demonstrate that the defect on virulence due to sustained parasite maintenance can be recovered either by *in vitro* or *in vivo* full differentiation to amastigote and back to promastigotes forms.

## Discussion

Visceral *Leishmania* infections studies have been the center of some controversy which can occasionally be traced back to the use of distinct *in vitro* promastigotes culture conditions. The plasticity of the *Leishmania* genome [20] is an important variable to consider when axenic promastigotes are used for *in vitro* or *in vivo* studies. Thus, parasite phenotypic plasticity allows it to adapt to the environment generating discrepancies between studies in different laboratories even when using the same *Leishmania* strain.

In the current work, we have started by investigating in our *in vitro* model of *L. infantum* infection the relation between the parasite development stage and its infectivity. The first step was to discard logarithmic parasites because they are not ultimately responsible for the infection [3], so we used a basic cell cycle analysis to discard multiplying parasites (less than 10% of total population are in S/G2 phase after the third day of culture). This data correlated clearly with basic morphological visualization. Stationary *L. infantum* cultures in day 5 and 9 induced higher BMMø infection rates than day 3 parasites. This difference in infectivity might translate the time frame required for becoming truly metacyclic parasites [21], which was also corroborated by the less amount of metacyclic recovered (data not shown). Since the presence of apoptotic parasites is essential for a virulent inoculum of *Leishmania* promastigotes [22], we decided to quantify the percentage of apoptotic and dead parasites in each case to remove this possible bias from our analysis. In fact, for all time frames tested, the differences in infectivity were not related to apoptotic or dead parasites in the non-purified or Ficoll-purified populations, with culture viability always higher than 90%.

Some authors described that *in vitro* maintenance for long periods constitute an important factor for the loss of virulence in *L. infantum* [14] and *L. major* [6] promastigotes. Nonetheless, this loss of virulence is a reversible phenomenon, since serial passages on susceptible mice allow the parasite to recover a virulence phenotype [23]. In the present study, we complemented the previous observations by comparing the impact of continuous *in vitro* culture on *Leishmania* promastigote virulence and also into the capacity of host macrophage manipulation. Our data clearly demonstrated a loss of *L. infantum* virulence related to the augmentation of *in vitro* culture periods although no modification was observed in the axenic promastigote growth behavior. This significant loss of infectivity was observed as soon as 105 days of successive (21 passages) culture and worsened with parasite maintenance in culture. In fact, 20 passages was the soonest time point where we could have a significant reproducible loss of infection. There is a grey area between passage 9 and passage 20 where we can have variation of infection in a manner that probably reflects the initial parasite inoculum recovered from the mammal. This was also observed after *in vivo* infection where we had a significant decrease in parasite burden after 21 and 31 passages, confirming the *in vitro* observations.

The percentage of metacyclic in a heterogeneous stationary-phase is an important factor in the parasite infectivity since they are significantly more infective than the non-purified population. We used a Ficoll density gradient methodology to enrich the percentage of metacyclic promastigotes [24]. Beyond the morphological changes, during the *Leishmania* spp. differentiation process, modifications also occur in gene expression and in the composition of parasite surface that help to characterize metacyclic promastigotes. Thus, we have evaluated the enrichment of metacyclic promastigotes in the Ficoll-purified fraction by microscopy (data not shown) and through qPCR analysis of the SHERP and histone H4 gene expression. The augmentation of SHERP gene expression in Ficoll-purification supported an enriched metacyclic population. The use of Ficoll-purified promastigotes abolished the differences found among the different passages. Yet, significant differences were found for P21 and P31, at 48 hours post-infection when facing an inflammatory stimulus, showing that the phenomenon of loss of virulence, although less prominent in the metacyclic-enriched parasites, was not restricted to the unpurified culture. Since the differences at the infection level were significant, we examined if there was a potential effect on the macrophage activation status. In the presence of a strong inflammatory stimulus, *L. infantum* is able to suppress certain LPS-derived pro-inflammatory cytokine responses in an active parasite-specific process while it augments the production of some anti-inflammatory cytokines (Silvestre et al, unpublished data). Indeed, the addition of LPS to *Leishmania* spp. infected cells was demonstrated to synergistically induce the secretion of the anti-inflammatory IL-10 cytokine in monocytes [25] and in macrophages [26]. The functional polarization of macrophages into IL-10 producers characterized as M2 cells [27] has been long understood to play a crucial role in the success of parasite infection process [28]. Our results demonstrated a growing defect of high passage parasites to modulate the LPS stimulatory effect. Furthermore, it is clear from the inflammatory profile depicted in Table 1 that metacyclic enriched fractions are always significantly more effective in abrogating a macrophage response to the inflammatory stimuli than their non-purified counterparts revealing the metacyclic parasites as a highly immunomodulatory population with a distinct profile from the non-purified population. There is a distinct and significant loss of immunomodulatory properties from P4 to P21 that becomes stable after P21. This loss of immunomodulatory properties seems reminiscent of phenomenon of transient gene expression similar to what happens under drug pressure [29], being lost upon the terminus of immunological pressure. Indeed, this might be happening in just a few passages of axenic culture. In an attempt to explain the loss of virulence mechanism, some authors referred to a reduction of metallo and cysteine peptidases activity, important for virulence, in *L. braziliensis* [30], [31] and in *L. amazonensis* [32] or mitochondrial defects [33] during long-term culture. However, others have been unable to detect any differences in the parasite enzymatic profile with long *in vitro* periods of cultivation [34], [35].

One can speculate that the overall loss of immunomodulatory properties over time, and in consequence loss of virulence might reflect a diminution of the number of metacyclic parasites in the population. Although the percentage of Ficoll-recovered promastigotes was quite similar among the three tested passages, these fractions do not constitute a pure metacyclic population, so we decided to complement older passage parasites with Ficoll recovered metacyclic promastigotes to access if the loss of virulence could be reverted by exogenous addition of metacyclic parasites from an early passage. No improvement in the overall infection was observed, although, when these same metacyclic parasites were added to an avirulent field strain, from which we were unable to recover metacyclics, there was an improvement on the infection.

Another possibility to explain the virulence loss was the possibility of a defective promastigote to amastigote differentiation. Our data clearly demonstrated that high passage number promastigotes displayed decrease capacity in differentiating, which was not correlated with decreased cellular viability. This incapacity translates into fewer parasites able to differentiate leading to a less capable population to face host cell response. Ultimately, this results in lower parasite burdens *in vitro* and *in vivo*. Moreover, these axenic amastigotes recovered after differentiation were morphologically indistinguishable and retained similar growth capacity (data not shown). To ultimately state and confirm the importance of promastigote to amastigote differentiation as a driving selective force for virulence, we showed that parasites passed through the amastigote stage, either *in vitro* or *in vivo*, revert the loss of virulence. This fact in conjunction with the remarkable loss of immunomodulatory properties leads to the early loss of virulence detected in our model.

We do not rule out metacyclogenesis related defects as a driving force for a virulence. In relation to metacyclogenesis it has been argued that a successful and complete differentiation is dependent on the presence of large amount of metacyclic promastigotes [36]. However, it is still not clear whether this process is an essential step in the differentiation *in vitro*, since procyclic promastigotes appear to differentiate with equal efficiency as metacyclics [37]–[40]. Indeed, our results with the naturally attenuated strain support this notion. Our data do not rule out that P21 or P31 metacyclic promastigotes could not display any sort of biochemical or protein expression defect that may impact the differentiation process. Similarly, we cannot reject the idea of longer periods of sustained cultured originating defective metacyclic cultures. However, during our study, the loss of virulence was related to a specific defect on promastigote to amastigote differentiation.

Overall, our data demonstrated that the loss of virulence is linked with decreased capacity to differentiate in amastigote forms, which may probably be originated from the absence of a complete life cycle. Therefore, special care must be taken when performing experiments with axenic *Leishmania* promastigotes. The systematic and rigorous control of *Leishmania* culture conditions should be considered as a keystone for each experimental protocol. The differences found in infectivity accompanied by disparate effects at the macrophage activation levels point to significant differences at biochemical and structural level, enlarging the effects of careless parasite maintenance to other experimental fields. This information is extremely relevant especially for those developing new drug and vaccine approaches. In such cases the immune response to the parasite is the essence of the experimental procedure.

## Supporting Information

Figure S1 **Cell cycle analysis and** ***in vitro*** **virulence of** ***L. infantum*** **recovered in distinct culture days.** *L. infantum* promastigotes were cultured at a 10^6^/ml. Each day, 2×10^6^ promastigotes were recovered and the cell cycle analyzed by PI staining (**A**). BMMø were incubated with non-purified CFSE labeled *L. infantum* promastigotes at a ratio of 1∶10 (cell/parasite). The percentage of infected cells was obtained by quantifying the number of CFSE-positive cells (**B**). Data were acquired at 4 and 24 hours post-infection in a FACScalibur cytometer and analysed by FlowJo software. Three independent experiments were performed; one representative experiment is shown. The mean and standard deviation are shown. *P<0,05, **P<0,01 statistical significance relatively to 3^rd^ day of parasite growth.
(TIF)

Figure S2 **Indirect quantification of metacyclic promastigotes in heterogenous and Ficoll-purified cultures by gene transcription analysis.** Transcription profile of SHERP and Histone H4 genes obtained by qPCR, for non-purified and Ficoll-purified promastigotes. Normalizations were made against the reference gene rRNA45. Three independent experiments were performed, each performed in duplicate; one representative experiment is shown. The mean and standard deviation are shown. *P<0,05.
(TIF)

Figure S3 **Long-term cultured** ***L. infantum*** **promastigotes show decrease capacity to modulate an inflammatory stimulus** ***in vitro*** **.** BMMø were submitted to LPS stimulation 4 hours after infection with non-purified promastigotes. The levels of IL-6 (**A**), TNF-α (**B**), IL-12p40 (**C**) and IL-10 (**D**) were quantified 24 hours post-infection on BMMø supernatants by ELISA. Three independent experiments were performed; one representative experiment is shown. The mean and standard deviation are shown. *P<0,05; **P<0,01; ***P<0,001 statistical differences relative to LPS unless depicted by a bar.
(TIF)

Figure S4 **Naturally attenuated** ***L. infantum*** **strain has a similar axenic growth in comparison to WT strain.** *L. infantum* growth curves were performed by neubauer chamber counting. The mean and standard deviation are shown.
(TIF)
