# Supplementary figures and images for: Impact of Continuous Axenic Cultivation in Leishmania infantum Virulence
Source: PLoS Negl Trop Dis. 2012 Jan 24;6(1):e1469. doi: 10.1371/journal.pntd.0001469 (PMC3265455; doi:10.1371/journal.pntd.0001469)

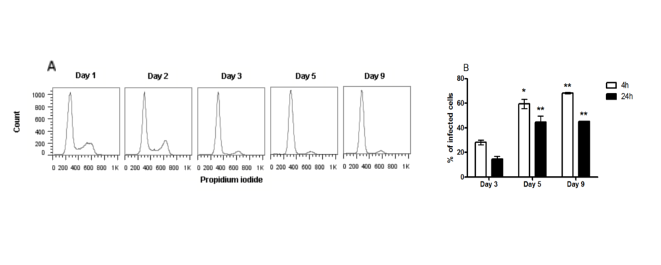

Supplement: Figure S1 — Cell cycle analysis and in vitro virulence of L. infantum recovered in distinct culture days. L. infantum promastigotes were cultured at a 106/ml. Each day, 2×10^6^ promastigotes were recovered and the cell cycle analyzed by PI staining (A). BMMø were incubated with non-purified CFSE labeled L. infantum promastigotes at a ratio of 1∶10 (cell/parasite). The percentage of infected cells was obtained by quantifying the number of CFSE-positive cells (B). Data were acquired at 4 and 24 hours post-infection in a FACScalibur cytometer and analysed by FlowJo software. Three independent experiments were performed; one representative experiment is shown. The mean and standard deviation are shown. *P<0,05, **P<0,01 statistical significance relatively to 3rd day of parasite growth. (TIF) [file pntd.0001469.s001.tif]

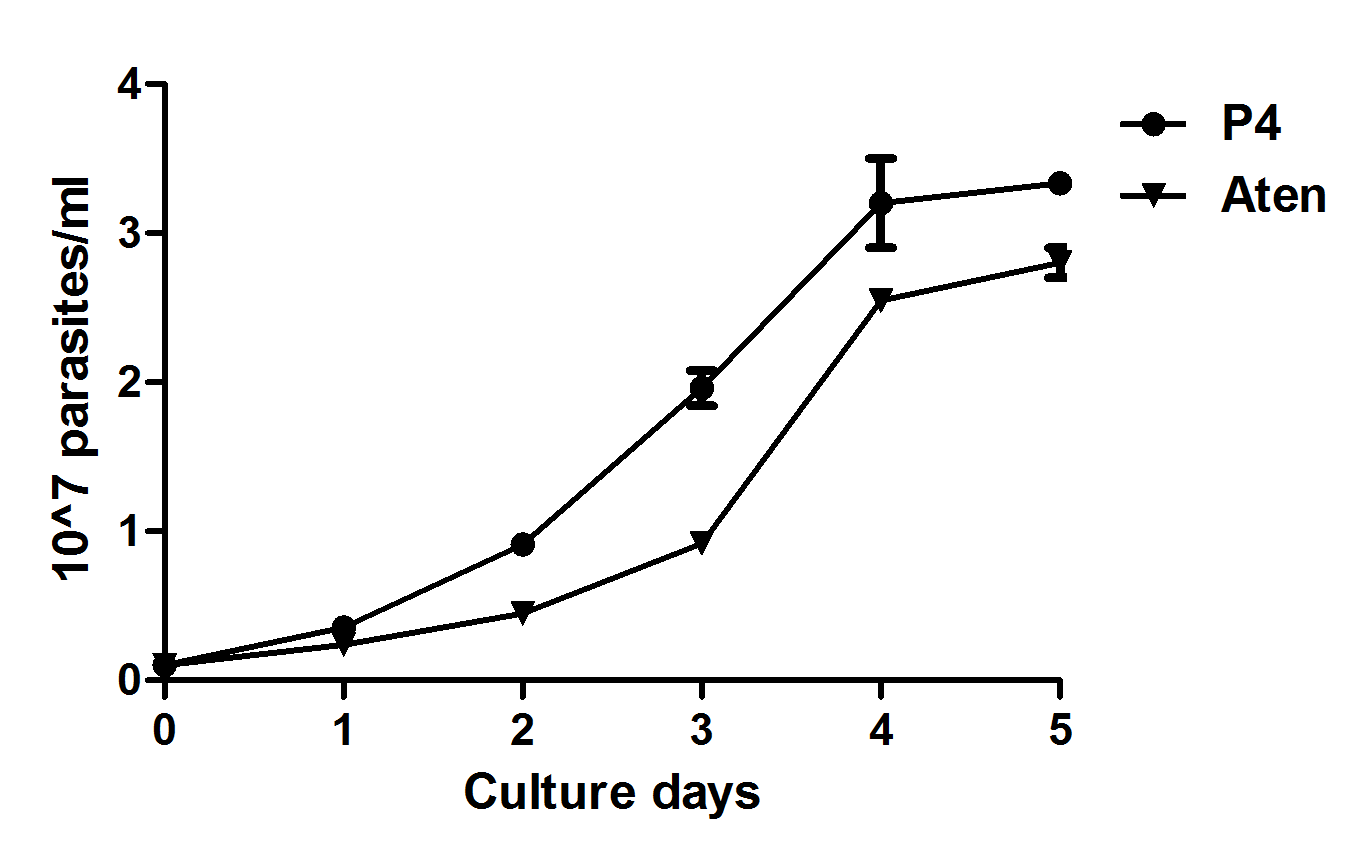

Supplement: Figure S4 — Naturally attenuated L. infantum strain has a similar axenic growth in comparison to WT strain. L. infantum growth curves were performed by neubauer chamber counting. The mean and standard deviation are shown. (TIF) [file pntd.0001469.s004.tif]
